# Androgen Receptor Copy Number Variation and Androgenetic Alopecia: A Case-Control Study

**DOI:** 10.1371/journal.pone.0005081

**Published:** 2009-04-02

**Authors:** Joanna E. Cobb, Stefan J. White, Stephen B. Harrap, Justine A. Ellis

**Affiliations:** 1 Department of Physiology, University of Melbourne, Melbourne, Victoria, Australia; 2 Murdoch Childrens Research Institute, Parkville, Victoria, Australia; Pasteur Institute, France

## Abstract

**Background:**

The functional polymorphism that explains the established association of the androgen receptor (*AR*) with androgenetic alopecia (AGA) remains unidentified, but Copy Number Variation (CNV) might be relevant. CNV involves changes in copy number of large segments of DNA, leading to the altered dosage of gene regulators or genes themselves. Two recent reports indicate regions of CNV in and around *AR*, and these have not been studied in relation to AGA. The aim of this preliminary case-control study was to determine if *AR* CNV is associated with AGA, with the hypothesis that CNV is the functional *AR* variant contributing to this condition.

**Methodology/Principal Findings:**

Multiplex Ligation-dependent Probe Amplification was used to screen for CNV in five *AR* exons and a conserved, non-coding region upstream of *AR* in 85 men carefully selected as cases and controls for maximal phenotypic contrast. There was no evidence of CNV in *AR* in any of the cases or controls, and thus no evidence of significant association between AGA and *AR* CNV.

**Conclusions/Significance:**

The results suggest this form of genomic variation at the *AR* locus is unlikely to predispose to AGA.

## Introduction

The importance of the gene encoding the androgen receptor (*AR*; NCBI NM_000044) in the development of androgenetic alopecia (AGA; OMIM %300710) is now well established. Our initial observation [1] of genetic association of *AR* with AGA has been replicated in three independent studies [2]–[4]. Although association with several single nucleotide polymorphisms (SNPs) has been demonstrated, none have proven causative. We have shown that known *AR* coding sequence polymorphisms such as triplet repeats are not associated with AGA [5], and mutation screening has identified no other relevant coding sequence mutations. Other forms of variation that might affect gene expression should now be considered.

Copy Number Variation (CNV) has been recognised recently as a common form of genomic variation [6]–[8] in which DNA segments (>1 kb) are altered in copy number compared to a reference genome. Recent reports have suggested that up to 12% of the genome is subject to CNV, accounting for at least 1% of genetic variation [6], [9].

CNV has been reported in the region of *AR* [6], [8] and SNPs can be co-inherited in linkage disequilibrium (LD) with CNV [6]. As CNV might result in variable copies of genetic regulatory regions or copies of the gene itself, such variation might underpin the tissue- and developmental stage-specific abnormalities in expression of *AR* seen in men predisposed to AGA.

We hypothesised that *AR* CNV could be associated with AGA. Therefore, our approach was to first search for evidence of CNV at the *AR* locus and then, if present, to test for association of CNV with AGA.

## Materials and Methods

### Subjects

The study was approved by the Ethics Review Committee of the Alfred Hospital Melbourne, and informed consent was obtained from each participant. Subjects were drawn from the population-based Victorian Family Heart Study cohort [10]. AGA phenotypes were gathered by way of direct assessment by trained observers and validated questionnaires [11]. Cases were 34 men aged 20–30 years (mean 27 years) with cosmetically significant baldness (Hamilton-Norwood types III vertex to VII, median type IV), and controls were 51 men aged 54–68 years (mean 56 years) with no indication of baldness (Hamilton-Norwood type I).

### MLPA analysis

**Figure 1:**
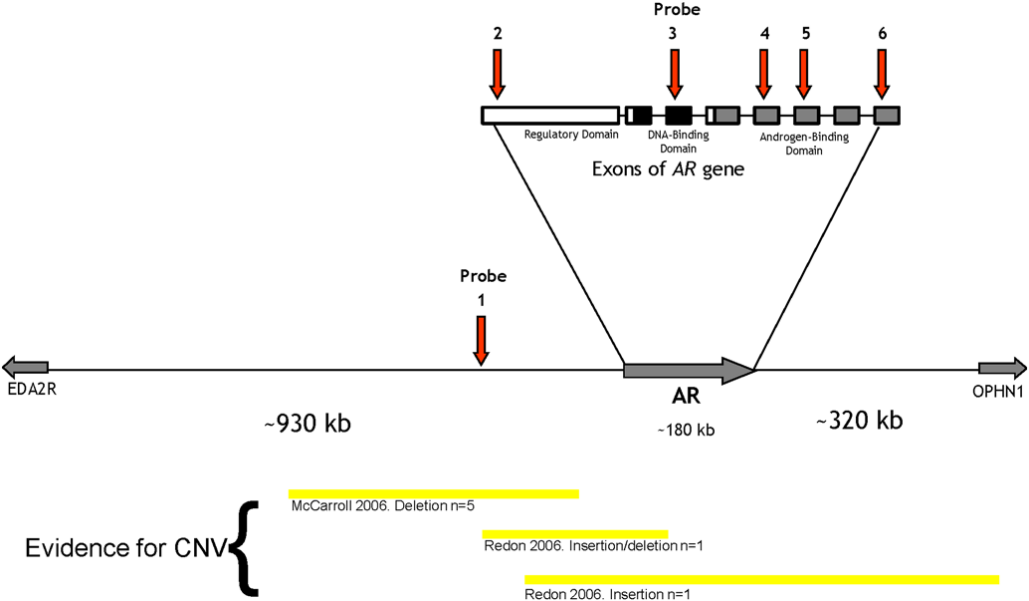
Location of the probes in relation to the androgen receptor. Diagrammatic representation of part of chromosome × showing the androgen receptor (*AR*) and the upstream (*EDA2R*) and downstream (*OPHN1*) genes. The relative locations of the upstream region (probe 1) and the five probes within the exons of *AR* are represented by the arrows. The *AR* gene exons are represented as boxes, as is the location of prior evidence of copy number variation (CNV) in this region.

Peripheral blood was collected from each subject, and DNA was extracted using standard phenol-chloroform methods. Multiplex Ligation-Dependent Probe Analysis (MLPA) was performed to detect the presence of CNV around *AR*. Six probes were selected to test *AR* exons and one upstream non-coding region for the presence of CNV (probes 1 – 6; Table 1 and Figure 1). Two further probes (7 and 8) in unlinked autosomal loci were used as reference point controls (Table 1). Peak heights were analysed for the eight probes for each DNA sample, as outlined in White *et al* [12]. Briefly, peak heights for each probe were standardized to control peaks and the median ratio for each probe normalised to 1.0 [12]. We set threshold values for duplication and deletions of 1.5 and 0.5 respectively [13]. All samples were analysed at least twice.

**Table 1:** Sequences and genomic locations of the probes.

Probe	Genomic Location	Sequence
**1**	ChrX: 66,481,078–66,481,135	5′ GGGTTCCCTAAGGGTTGGAGACTTAAGGGTACATAATAATGGGCAGTGG 3′
		5′ ATTATGTGGACACCACAATTTGGAAGGGTCTAGATTGGATCTTGCTGGC 3′
**2**	ChrX: 66,681,149–66,681,198	5′ GGGTTCCCTAAGGGTTGGAGCTTCAGCACTGCAGCCACGACCC 3′
		5′ GCCTGGTTAGGCTGCACGCGGAGAGATCTAGATTGGATCTTGCTGGC 3′
**3**	ChrX: 66,822,629–66,822,690	5′ GGGTTCCCTAAGGGTTGGACCGAAGGAAAAATTGTCCATCTTGTCGTCT 3′
		5′ TCGGAAATGTTATGAAGCAGGGATGACTCTGGTCTAGATTGGATCTTGCTGGC 3′
**4**	ChrX: 66,854,045–66,854,113	5′ GGGTTCCCTAAGGGTTGGAGCTTCCGCAACTTACACGTGGACGACCAGATGGCT 3′
		5′ GTCATTCAGTACTCCTGGATGGGGCTCATGGTGTCTAGATTGGATCTTGCTGGC 3′
**5**	ChrX: 66,858,400–66,858,453	5′ GGGTTCCCTAAGGGTTGGAGTACCGCATGCACAAGTCCCGGATGTA 3′
		5′ CAGCCAGTGTGTCCGAATGAGGCACCTTCTAGATTGGATCTTGCTGGC 3′
**6**	ChrX: 66,860,242–66,860,342	5′ GGGTTCCCTAAGGGTTGGACGAGAGAGCTGCATCAGTTCACTTTTGACCTGCTAATCAAG 3′
		5′ TCACACATGGTGAGCGTGGACTTTCCGGAAATGATGGCAGAGATCTAGATTGGATCTTGCTGGC 3′
**7**	Chr16: 3,772,843–3,772,914	5′ GGGTTCCCTAAGGGTTGGACCAGCTAGTGGAATTCAAAACACAATTGGTTCTGTTGGCACA 3′
		5′ GGGCAACAGAATGCCACTTCTTTAAGTAACTCTAGATTGGATCTTGCTGGC 3′
**8**	Chr22: 39,857,407–39,857,482	5′ GGGTTCCCTAAGGGTTGGACCAACCTAAGCACTGTTAGTCAGATTGATCCCAGCTCCAT 3′
		5′ GAAAGAGCCTATGCAGCTCTTGGACTACCCTATCATCTAGATTGGATCTTGCTGGC 3′

Listed are the *AR* exonic and non-coding region probes (1–6), and control probes (7 and 8). Probes 7 and 8 were used as controls to normalize data and were designed in regions with no known CNV. Genomic location data based on human reference sequence (NCBI Build 36.1).

## Results

**Figure 2:**
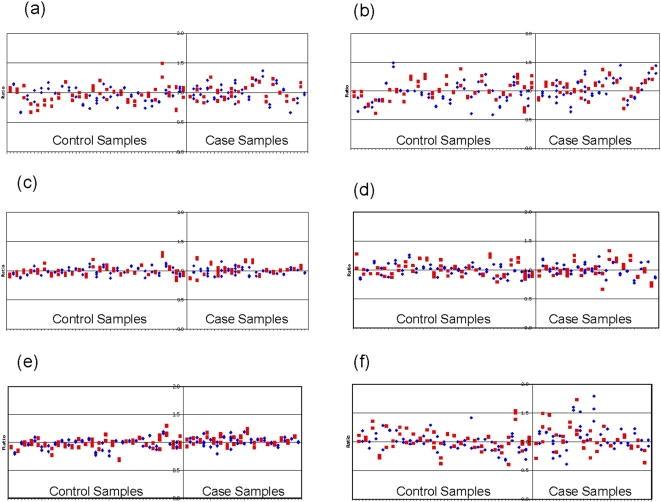
(a-f): Normalised ratios for both case and control samples across the six test probes. Each plot includes at least two replicates per sample represented by the same symbol, with the symbols alternating between each sample. Samples were normalised using two control probes (see Table 1). Normal range 0.5–1.5.

Figure 2 shows data for each of the six *AR* test probes in all case-control subjects. The standard deviation for each of the six *AR* test probes was within 15%, except for probes 2 and 6 which were slightly higher, possibly suggesting less reliable performance of the probes. Nevertheless, across the remaining samples, good coverage of the *AR* region was achieved by the higher performing probes, and the results provide no evidence of CNV in this region, either as deletion or duplication, in cases or controls.

## Discussion

This study examined CNV in and around *AR* and their potential relationship with AGA. CNV may contribute to differences in gene expression through duplications or deletions of genes or gene regulatory elements [14], and may in part account for genetic predisposition to common complex conditions such as AGA. However, we found no evidence of CNV in the regions of the *AR* that we examined. Insofar as our original hypothesis, we could therefore find no evidence of association of CNV with AGA.

Although data generated by probes 2 and 6 showed relatively high standard deviations, casting doubt on their reliability, the *AR* gene was well covered by the remaining probes. The lack of evidence for CNV at this locus supports the findings of a very recent study by McCarroll *et al* which has demonstrated a lack of CNV in the *AR* coding regions [15].

This study examined a locus on chromosome × in males, meaning that if changes in CNV dosages were detected, they would be expected to be at either a 0 (deletion) or 2 (duplication) level. Figure 2 demonstrates that the results for some samples showed normalised ratios of around 0.5 or 1.5, consistent with possible mosaicism within the cells of the blood samples from which the DNA was extracted. CNV mosaicism has been demonstrated in blood samples in monozygotic twins [16], and it is known that CNV may be variable between cell types within the same individual. Although the results presented here indicate no evidence of *AR* CNV in DNA derived from lymphocytes, we cannot rule out CNV around the *AR* in DNA contained in the cells of the hair follicles themselves.

Our preliminary investigation did not thoroughly investigate the downstream non-coding flanking region and other more distant sites from *AR*. However, we focussed on a region that we have already shown to be strongly associated with AGA and it would be likely that causative CNV, if they exist, would be in the region examined. Sample sizes used were relatively small, however power is augmented by the fact that association of AGA with *AR* is well-established and that the cases and controls were selected to represent high phenotypic contrast.

In the absence of evidence of *AR* CNV involvement in AGA, other forms of non-coding *AR* sequence variation, and also epigenetic mechanisms at this locus, should be considered in clarifying the role of *AR* as a genetic risk locus for AGA.
